# Characterization of genome-wide transpositions induced by colistin exposure in multi-drug-resistant *Klebsiella pneumoniae*

**DOI:** 10.1128/aac.01574-24

**Published:** 2025-05-19

**Authors:** Sahaya Glingston Rajakani, Basil Britto Xavier, Ngoc Minh Nguyen, Qiang Lin, Anouk Braspenning, Nienke L. Plantinga, Bastiaan H. J. Wittekamp, Olympia Zarkotou, Rob Van Houdt, Youri Glupczynski, Spyros Pournaras, Marc J. M. Bonten, Surbhi Malhotra-Kumar

**Affiliations:** 1 Laboratory of Medical Microbiology, Vaccine and Infectious Disease Institute, Universiteit Antwerpen 198686, Antwerp, Belgium; 2 Julius Center for Health Sciences and Primary Care, University Medical Center Utrecht 168086 , Utrecht, The Netherlands; 3 Department of Microbiology, Tzaneio Hospital, Piraeus, Greece; 4 Microbiology Unit, Belgian Nuclear Research Centre (SCK CEN) 74873, Mol, Flanders, Belgium; 5 Laboratory of Clinical Microbiology, Attikon University Hospital, Medical School, National and Kapodistrian University of Athens 68993, Athens, Greece; 6 European Clinical Research Alliance on Infectious Diseases (Ecraid), Utrecht, the Netherlands; Shionogi Inc., Florham Park, New Jersey, USA

**Keywords:** antibiotic resistance, insertion sequence elements, colistin, muti-drug resistant Klebsiella penumoniae

## Abstract

Colistin is one of the last-line antibiotics against multi-drug-resistant (MDR) gram-negative pathogens, such as *Klebsiella pneumoniae*. Using long-read sequencing, we observed remarkable genome-wide transposition events in MDR *K. pneumoniae* exposed to colistin in patients receiving treatment for respiratory infections or as part of selective decolonization strategies and further confirmed these on *in vitro* selection experiments. These data add yet another dimension to the role of antibiotics in mediating specific processes in bacteria beyond antibiotic resistance.

**CLINICAL TRIALS:**

This study is registered with ClinicalTrials.gov as NCT02208154.

## INTRODUCTION

Insertion sequence (IS) elements are the most abundant transposable elements with a size of less than 2.5 kb, which can cause small- to large-scale mutations and genome rearrangements in both laboratory and clinically isolated bacteria (1, 2). Such IS element-induced mutations and genome rearrangements are one of the major forces of genome evolution that facilitate the spread of antibiotic resistance within and across bacterial species (1, 2).

Colistin is one of the last-line antibiotics utilized against infections caused by multi-drug-resistant (MDR) *Klebsiella pneumoniae* (3, 4) and is categorized by the World Health Organization as of “very high importance for human medicine” (5). Colistin is positively charged, thus binding to the negatively charged phosphate groups of lipid A and leading to a competitive displacement of the divalent cations of calcium (Ca^2+^) and magnesium (Mg^2+^). This effect compromises the three-dimensional structure of the lipopolysaccharide (LPS) and allows colistin to pass through the permeabilized outer membrane in a self-promoted uptake mechanism. One of the most common mechanisms of colistin resistance reported in *K. pneumoniae* is the IS element-induced disruption or interruption of the *mgrB* gene, which encodes a negative regulator of the two-component system, PhoQ-PhoP, thus resulting in its constitutive expression and in LPS modifications that allow cell division and growth in the presence of colistin (6, 7). Furthermore, tetracycline pressure has also been shown to drive antibiotic resistance gene duplications through mobile genetic (IS) transposition (8). These examples highlight IS element-mediated disruption or duplication of specific chromosomal genes as a common and important mechanism of antibiotic resistance emergence in bacteria under antibiotic pressure. However, the non-specific, genome-wide effects of antibiotic use on the bacterial genome, if any, are not well-explored.

Hence, this study strives to explore the genome-wide effects observed in MDR *K. pneumoniae* strains exposed to colistin either in patients undergoing treatment for respiratory infections or as part of preventive selective decolonization strategies in intensive care unit (ICU) patients involving colistin. We examined 12 pairs of MDR *K. pneumoniae* strains (each pair comprising one colistin-susceptible strain and one colistin-resistant strain) isolated from patients. Furthermore, a colistin-resistant strain was experimentally generated *in vitro* using only colistin to validate the findings.

When comparing the presence of IS elements between the colistin-resistant strains isolated after colistin exposure and the corresponding colistin-susceptible strains in patients receiving colistin, we observe on average a 33% (range: 12% – 121%) increase in the number of IS elements in the colistin-resistant strains in 8/10 clinically isogenic pairs studied here (Table 1; resistance profiles of MDR *K. pneumoniae* strains used in the study are detailed in Table S1).

**TABLE 1:** Summary of strain characteristics and increase in the number of IS elements after colistin exposure in clinically isogenic *K. pneumoniae* isolated from patients before and after colistin use^*f*^

Strain no.	Strain ID	Bodysite	Intervention	Culture type	Date of isolation	Country	ST type	Colistin phenotype	Time between isolation of strain in days	No. of IS elements	Percent change in IS elements
1	FE0883S	Rectum	SDD^*a*^	Surv^*c*^	Oct 15	Spain	437	S	0	21	
	FE0883R	Rectum	SDD	Surv	Oct 15	Spain	437	R	12	30	43%
2	FE1279S	Rectum	SOD^*b*^	PPS^*d*^	Mar 16	Spain	147	S	0	34	
	FE1279R	Rectum	SOD	Surv	Mar 16	Spain	147	R	7	38	12%
3	IT0374S	Aspirate	SOD	Surv	Jun 15	Italy	101	S	4	30	
	IT0374R	Aspirate	SOD	Surv	Aug 15	Italy	101	R	46	37	23%
4	IT0385S	Aspirate	SOD	Clinical	Aug 15	Italy	101	S	30	35	
	IT0385R	Aspirate	SOD	Surv	Sep 15	Italy	101	R	49	41	17%
5	UZ7021S	Rectum	SDD	Clinical	Feb 16	Belgium	70	S	0	17	
	UZ7021R	Rectum	SDD	Surv	Feb 16	Belgium	70	R	2	19	12%
6	LB2648S	Aspirate	SOD	Clinical	Oct 15	Belgium	17	S	0	5	
	LB2648R	Aspirate	SOD	Surv	Nov 15	Belgium	17	R	29	6	20%
7	IT0649S	Rectum	SOD	Surv	May 16	Italy	409	S	0	19	
	IT0649R	Rectum	SOD	PPS	Jun 16	Italy	409	R	42	18	−5%
8	BC0799S	Aspirate	SOD	Clinical	Sep 16	Spain	405	S	0	39	
	BC0799R	Aspirate	SOD	Surv	Feb 17	Spain	405	R	151	36	−8%
9	3319S	Blood	colistin	Infection	Apr 09	Greece	258	S	0	34	
	3359R	Urine	colistin	Infection	May 09	Greece	258	R	31	75	121%
10	116S	CVC	colistin	Infection	Feb 10	Greece	383	S	0	18	
	117R	Blood	colistin	Infection	Apr 10	Greece	383	R	30	21	17%
11	AN0157S	Aspirate	baseline	Surv	Mar 14	Belgium	11	S	0	25	
	AN0157R	Aspirate	baseline	Clinical	Mar 14	Belgium	11	R	0	22	NA^*e*^
12	IT0132S	Rectum	baseline	Surv	Oct 14	Italy	101	S	0	45	
	IT0132R	Rectum	baseline	Surv	Oct 14	Italy	101	R	18	45	NA^*e*^
13	3319S-2R	*In vitro*	NA	Laboratory	Mar 13	Belgium	258	R	0	61	79%
	3319S-4R	*In vitro*	NA	Laboratory	Mar 13	Belgium	258	R	0	53	56%
	3319S-10R	*In vitro*	NA	Laboratory	Mar 13	Belgium	258	R	0	67	97%

^*a*^ SDD: selective digestive tract decontamination.

^*b*^ SOD: selective oropharyngeal decontamination.

^*c*^ Surv: surveillance.

^*d*^ PPS: point prevalence survey.

^*e*^ NA: not applicable, as patients during baseline did not undergo decolonization and were not exposed to colistin.

^*f*^ Strain pairs 11 and 12 were collected from patients not exposed to colistin and were used as controls.

To confirm that colistin-resistant strains were derived from the susceptible isolates, we analyzed the plasmid profiles of each strain and performed a single-nucleotide polymorphism (SNP) distance matrix analysis to assess the level of genetic relatedness between the colistin-susceptible and -resistant strain pairs.

For most pairs (11 out of 13), the antimicrobial resistance (AMR) profiles were either identical between the colistin-susceptible (Col-S) and -resistant (Col-R) strains or showed minor variations in AMR gene content (data not included in the manuscript). Such subtle differences in the resistome content may be attributed to the small size of the contigs or insufficient sequencing depth. In two pairs (Fe0883S/Fe0883R and 3319/3359R), where the colistin-susceptible and -resistant strains displayed very different AMR gene profiles, an acquisition of plasmids associated with carbapenemase resistance genes (OXA-48 on an IncL plasmid and KPC-2 on an IncX3 plasmid) appears evident.

To further study the genetic relationship between colistin-susceptible and -resistant strain pairs, we performed cgSNP analysis (Parsnp) using a complete set of 713 clinical *K. pneumoniae* strains (RGNOSIS-set) with calculated distance metrics (snp-dists) and a predefined cut-off for clonality of less than 20 SNPs (complete data set not shown). Such a low SNP distance would indicate recent evolutionary divergence, supporting the hypothesis that resistance emerged through genetic modifications within the susceptible lineage. The SNP distance between the strain pairs is shown in Table S1.

To further analyze the genetic modifications caused by the IS elements, one clinically derived isogenic pair was examined in detail: 3319S (pre-exposure, colistin-susceptible) and 3359R (post-exposure, colistin-resistant). In addition to the disruption of the *mgrB gene* by IS*Kpn26*, which confers colistin resistance, several additional IS elements were found to be located at the 5′ or 3′ end of multiple other chromosomal genes in 3359R, including genes encoding for choline transporter, mercuric transporter protein, and ABC transporter ATP-binding cassette protein (data not shown). Overall, we identified a >twofold increase in the IS element content of the resistant strain following colistin exposure compared to its corresponding susceptible counterpart (3319S: *n* = 34; 3359R: *n* = 75) (Table 1).

**Fig 1:**
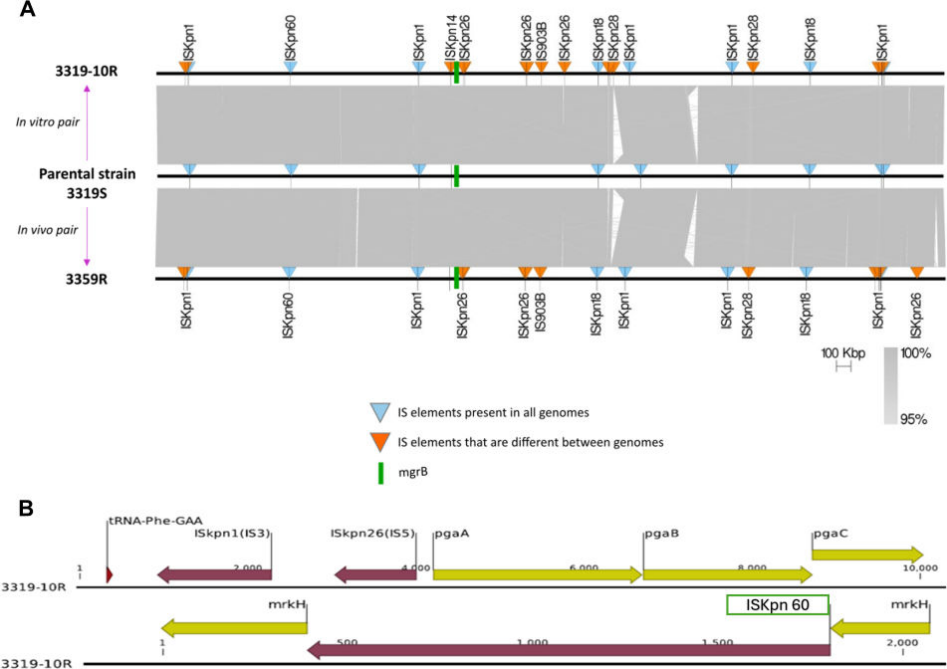
Condensed whole-genome synteny map of MDR *K. pneumoniae* strains isolated pre- (3319S) and post-colistin treatment (3359R) from a Greek patient and a derivative strain from 3319S (3319-10R, colistin MIC 64 mg/L) generated *in vitro* by passaging 3319S under colistin pressure (**A**). Visualization of selected genomic regions of strain 3319-10R displaying the IS*Kpn1* and IS*Kpn26* insertions in the promoter region of the *pga* operon (top) and the IS*Kpn60* insertion in the PilZ-domain encoding region of *mrkH* (bottom). Both genome regions are associated with biofilm formation in *K. pneumoniae* (**B**).

**Fig 2:**
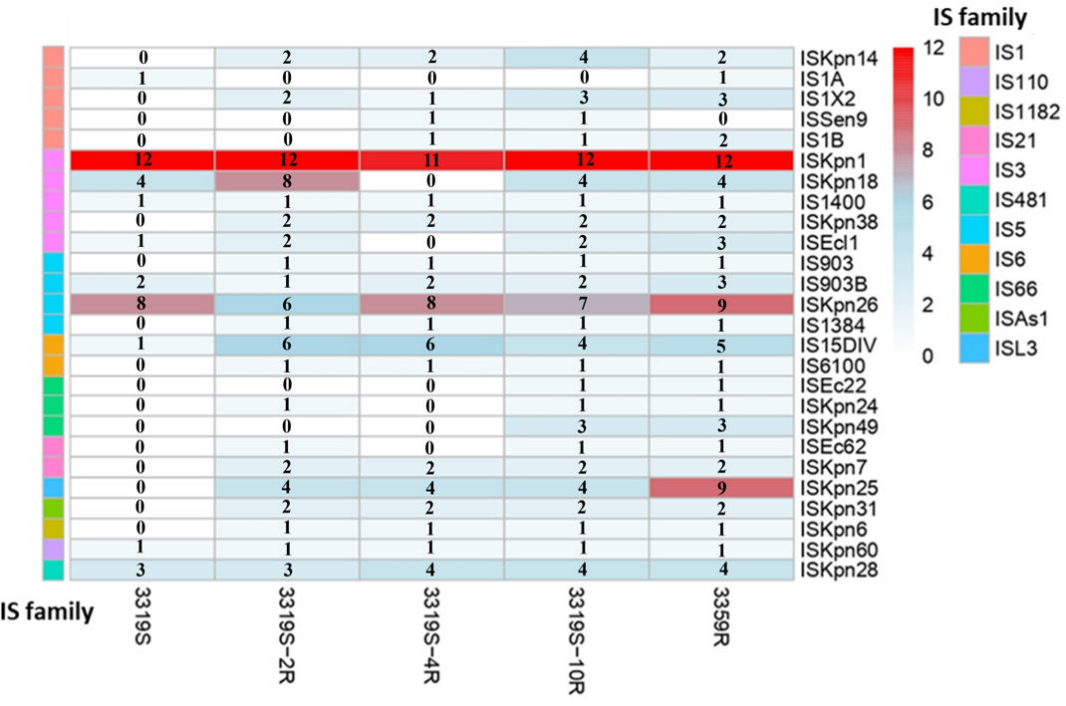
Heatmap demonstrating IS element amplification in the colistin-resistant *K. pneumoniae* strain 3359R compared to the colistin-susceptible 3319S and strains generated by passaging 3319S under colistin pressure (3319-2R, colistin MIC 64 mg/L; 3319-4R, colistin MIC 64 mg/L; 3319-10R, colistin MIC 64 mg/L).

To understand the precise impact of colistin pressure in causing the increased IS transposition observed in clinical isolates, an *in vitro* evolution experiment was conducted using the colistin-susceptible clinical strain, 3319S. Strain 3319S was passaged multiple times under increasing colistin pressure, after which three random *in vitro*-generated colistin-resistant colonies were isolated (3319S-2R, 3319S-4R, and 3319S-10R) with a colistin MIC of 64 mg/L. To study the stability of the acquired resistance, all resistant clones were passaged on colistin-free media and showed stable and inherited resistance (MIC 64 mg/L) on MIC screenings for more than 5,760 generations. As observed in the clinical colistin-resistant strain 3319R, the three *in vitro* generated resistant strains were also found to harbor almost double the number of IS elements (3319S-2R [*n* = 61], 3319S-4R [*n* = 53], and 3319S-10R [*n* = 67] compared to the parent strain, 3319S (*n* = 34) (Table 1) (Fig. 1A). Simultaneously, when 3319S was serially passaged in CAMHB without colistin for three consecutive days, no increase in the number of IS elements was observed. The IS elements, more specifically IS*15DIV* (a minor variant of IS*26*) from the IS*6* family, IS*Kpn25* from the IS*L3* family, and IS*Kpn14* and IS*1 × 2* from the IS*1* family, were identified to have multiple-fold increased copy numbers after colistin exposure (Fig. 2). Compared to 3319S, the genome of the *in vitro* generated 3319-10R showed unique IS-mediated interruptions, including (i) IS*Kpn14* (IS*1* family) inserted in *mgrB* at the 21st nucleotide, (ii) IS*Kpn14* (IS*1* family) inserted in the intergenic region of genes coding for a YobH and YebO family protein, (iii) IS*600* (IS*6* family) inserted in the promoter region of a gene coding for a thermal regulator protein, (iv) IS*903* (IS*5* family) inserted in the promoter region of the lactose proton symport-coding gene, and (v) IS*Kpn14* (IS*3* family) identified as disrupting the gene encoding the phenylacetic acid degradation protein. We also identified disruptions in genes involved in biofilm formation in *K. pneumoniae* (i.e., by IS*Kpn26* and IS*Kpn1* in the promoter region of the *pga* gene cluster and ISEc21 (IS*110* family) in the PilZ domain-encoding region of *mrkH*) (Fig. 1B) (9, 10). However, 3319S to 3359R belong to sequence type (ST) 258 comprising poor biofilm formers (11). This did not allow us to observe any differences in biofilm-related phenotypes via static biofilm assays with respect to these genetic changes in 3319S (OD_492_: −0.00775) and 3359R (OD_492_: −0.00454).

The colistin-resistant phenotype of 3319-10R could be attributed to the insertion of IS*Kpn14* 21 bases upstream of the start codon of *mgrB*. The origin of IS*Kpn14* could be traced to the scaffolds belonging to plasmid pKpQIL-GR-3319. IS*Kpn14* harbors two intact open reading frames, *insA* and *insB*, necessary for transposase function and transposes by a copy-paste mechanism into the *mgrB* gene (12). Furthermore, a comparative WGS analysis of *in vitro* serially passaged 3319S without colistin pressure for the same number of generations confirmed that IS*Kpn14* did not spontaneously transpose into *mgrB* or elsewhere, further confirming colistin as the trigger for transposition from the pKpQIL plasmid.

The pKpQIL plasmid harbors two IS elements, IS*Kpn14* and IS*Kpn25*, both of which have insertion sites in the *mgrB* gene in *K. pneumoniae*. To identify the preferred IS element interrupting *mgrB*, we performed population analysis profiling on the *in vitro* colistin-resistant clones generated from 3319S. The *in vitro* clones (*n* = 140, 20 from each concentration) selected from increasing concentrations of colistin (2, 4, 8, 12, 16, 32, and 64 mg/L) showed that interruption by IS*Knp14* (780 bp) was highly prevalent at every concentration, while interruptions by IS*Kpn25* were identified at a very low prevalence among the *in vitro* generated colistin-resistant clones of 3319S. On the contrary, IS*Kpn25* interruption of *mgrB* observed in the clinical colistin-resistant 3359R was the most probable cause of the *in vivo* emergent colistin-resistant phenotype.

With the help of long- and short-read hybrid analyses, we provide clear *in vivo* evidence, further confirmed by controlled *in vitro* experiments, that colistin acts as an evolutionary driver of rapid and massive IS-mediated genomic changes in MDR *K. pneumoniae* by mediating transposition of IS elements harbored on both plasmids and the chromosome.

A few limitations of the study are as follows: first, IS element transposition as a process is potentially affected by the concentration of colistin to which the organism is exposed; the *in vivo* strain exposure to colistin is not homogenous, as patients received the antibiotic for treatment or for selective oral or digestive decontamination (see methods for more details) at varying concentrations; second, the mode of transposition of IS elements can vary (cut and paste versus copy and paste), thereby impacting the total increase in IS elements post-colistin exposure; these factors probably account for the variation in IS proliferation across various ST types of *K. pneumoniae* exposed to colistin *in vivo*; third, isolate 3319 (IS number increased by 121%) clearly differed from the majority of the clinical isolates that only displayed increases of up to 20%, with only two colistin-exposed strains exceeding this threshold. This may have disproportionately inflated the overall significance of the findings, necessitating cautious interpretation of the data.

Nonetheless, this phenomenon observed across strains and STs of clinically relevant MDR *K. pneumoniae* associated with hospital infections has strong clinical implications. IS expansion can lead to pseudogene formation during pathoadaptation by intrachromosomal recombination or by generating deletions, the latter resulting in loss of the targeted gene, as well as of the IS elements themselves (1). Such genome reductions can result in streamlined ‘fitter’ genomes with increased bacterial pathogenicity and virulence (1). IS expansions in bacterial genomes have been reported previously, and analysis on sequenced genomes has shown it to be a major determinant in generating several of the present-day pathogens, such as *Bordetella pertussis* and *Bordetella parapertussis*, *Yersinia pestis*, *Enterococcus faecium*, *Mycobacterium ulcerans*, and many others. Specifically, in *Bordetellae*, large-scale genome rearrangements and deletions associated with IS expansion that improved the bacterium’s ability to combat host defenses were demonstrated to be a consequence of loss, not gain, of function, and differences in virulence were potentially related to loss of regulatory or control functions (13). However, such studies on IS element-related evolutionary trajectories in bacteria span over several decades. In contrast, the genome-wide changes effected by colistin are condensed in terms of evolutionary timescales and, importantly, are stable despite the lack of colistin pressure.

Through our work, we demonstrate that MDR *K. pneumoniae* exposed to colistin in patients being treated for respiratory infections or undergoing selective gut decolonization show remarkable genome-wide transposition events evidenced by an increase in the total number of IS elements present in their genome post-colistin exposure. We further confirm these data using *in vitro* evolution experiments with and without colistin as the sole pressure to identify its causative role by eliminating those of other variables potentially affecting transposition in the *in vivo* exposed *K. pneumoniae*. These data add yet another dimension to the role of antibiotics in mediating specific processes beyond antibiotic resistance.

Twelve pairs (colistin-susceptible and colistin-resistant) of ‘clinically isogenic’ *K. pneumoniae* strains belonging to the same ST for each patient were utilized in this study (Table 1). Of the 12 pairs, 10 originated from screening or clinical specimens (rectal swabs, endotracheal aspirates) of patients enrolled in the multi-center clinical trial, Resistance in Gram-Negative Organisms: Studying Intervention Strategies (R-GNOSIS) (ClinicalTrials.gov NCT02208154; EU-FP7 R-GNOSIS, 2014 – 2017). Eight of the 10 strain pairs were collected in Belgium (*n* = 2), Italy (*n* = 3), or Spain (*n* = 3) from mechanically ventilated ICU patients subjected to either selective oropharyngeal decontamination (SOD) or selective digestive tract decontamination (SDD), two infection prevention strategies that use colistin along with other anti-infective agents (14). During SOD and SDD, an oropharyngeal paste, which contained 0.19 million units of colistin sulfate, 10 mg of tobramycin sulfate, and 0.1 million units of nystatin per dosage (0.5 g), was used. Additionally, in SDD, a gastrointestinal suspension was used, which contained 1.9 million units of colistin sulfate, 80 mg of tobramycin sulfate, and 2.0 million units of nystatin per dosage (10 mL through nasogastric tube). The remaining two of the 10 pairs (one from Belgium and one from Italy) were obtained from screening/clinical samples of patients who did not undergo SOD or SDD (Baseline, Table 1). Finally, two strain pairs were isolated from clinical samples (blood, urine, and central venous catheter) of two Greek patients who underwent colistin treatment at the Tzaneio General Hospital, Piraeus, Greece (Table 1). The first pair consists of sequentially isolated colistin-sensitive (3319S from blood culture) and -resistant (3359R from urine culture) *K. pneumoniae* ST258, which were obtained from an ICU patient who underwent 13 days of colistin treatment after the isolation of the colistin-sensitive 3319S and prior to isolation of the colistin-resistant 3359R. The second pair (116S from central venous catheter culture and 117R from blood culture), which belongs to *K. pneumoniae* ST383, was obtained from a patient in the medical ward who underwent 30 days of colistin treatment after the isolation of the colistin-sensitive 116S and prior to isolation of the colistin-resistant 117R.

Minimum inhibitory concentrations (MICs) to colistin of all investigated strains (*n* = 24) were initially determined at the local sites either by E-test (bioMérieux, Marcy l’Etoile, France) or by a semi-automated or automated testing method: BD Phoenix (BD Diagnostics, Le Pont de Claix, France), Sensititre (Thermo Fisher Scientific, Waltham, MA, USA), Vitek (bioMérieux, Marcy l’Etoile, France), or MicroScan (Beckman Coulter, San Diego, CA, USA). Colistin susceptibility was subsequently confirmed at our laboratory for all strains by broth microdilution (MICRONAUT MIC-Strip Colistin, MERLIN Diagnostika GmbH, Berlin, Germany). Strains were classified as susceptible (MIC ≤ 2 mg/L) or resistant (MIC > 2 mg/L) to colistin based on the European Committee on Antimicrobial Susceptibility Testing breakpoints (breakpoint tables for interpretation of MICs and zone diameters, version 10.0, 2020). Two colistin-susceptible strains, *Escherichia coli* ATCC 25922 (MIC: 0.25–2 mg/L) and *Pseudomonas aeruginosa* ATCC 27853 (MIC: 0.5–4 mg/L), and two colistin-resistant strains, *K. pneumoniae* 08400 (MIC: 64 mg/L) and *E. coli* NCTC 13846 *mcr-1-*positive (MIC: 4 mg/L), were used as controls.

A colistin-susceptible *K. pneumoniae* strain (3319S, MIC = 1 mg/L, clinical pair #8, Table 1) was subjected to serial passaging under sub- and supra-MIC concentrations of colistin (0.5, 0.75, 1, 2, and up to 64 mg/L) to induce colistin resistance *in vitro*. Briefly, the 3319 S *K*. *pneumoniae* strain was grown overnight in cation-adjusted Mueller Hinton broth (CAMHB) (BD Diagnostics, Le Pont de Claix, France) with 0.5 mg/L colistin (Sigma-Aldrich, St. Louis, MO, USA). Subsequently, 200 µL of the overnight culture was inoculated into 2 mL of CAMHB containing 0.75 mg/L colistin. This procedure was repeated with a concentration of 1 mg/L colistin in the third passage and twofold increasing concentrations of colistin in the passages thereafter up to 64 mg/L. Among the generated resistant clones, three (3319S-2R, 3319S-4R, and 3319S-10R) were isolated from plates with 64 mg/L colistin. Their MICs were determined using broth microdilution and further subjected to whole-genome sequencing (WGS, see below section).

To assess the stability of the *in vitro* generated colistin resistance, these clones (3319S-2R, 3319S-4R, and 3319S-10R) were further passaged on antibiotic-free cation-adjusted Mueller-Hinton agar (CAMHA) (BD Diagnostics, Le Pont De Claix, France) for 160 days (approximately 5,760 generations). The stability of the acquired colistin resistance was evaluated every 5 days (approximately 200 generations) by MIC determination.

*In vitro* resistant clones obtained from the 3319S strain were further screened by population analysis to determine the frequency and type of *mgrB* modifications that led to colistin resistance. Briefly, 20 colonies were randomly picked from each colistin concentration (2, 4, 8, 12, 16, 32, 64 mg/L) and sub-cultured on CAMHA plates. A total of 140 colonies were picked from different generations (G5, G6, G7, G24, G34, G55, G68, G75, G84, G96, G107, G107, G117, G118, G132, G151, G161), and *mgrB* modifications were determined by PCR. In-house developed primers were used to identify modifications or interruptions in *mgrB*, Fw: 5′ TCTGAGTCCACAGCAACAGG 3′ and Rev: 5′ AGAGAAACTCCACCACTTTA 3′. For identification of *mgrB* promoter interruptions, *mgrB*-F1 5′ ATAACACCCCATAACCGTC 3′ and *mgrB*-R1 5′ AGAGAAACTCCACCACTTTA 3′ were used.

Briefly, for Illumina sequencing, genomic DNA from all 27 strains (Table 1) was isolated using the Master Pure Complete DNA and RNA Purification Kit (Epicentre, Madison, WI, USA) according to the manufacturer’s protocol. DNA concentration was checked with Qubit DNA and RNA assay (Life Technologies, Thermo Fisher Scientific, Inc., CA, US).

For long-read sequencing, genomic DNA from the clinically isogenic pair, 3319S-3359R, and its *in vitro* generated resistant clone, 3319-10R, was isolated using the Qiagen MagAttract HMW Kit (Qiagen, Hilden, Germany) according to the manufacturer’s protocol. Isolated genomic DNA was sheared using Covaris G-tubes to fragment size distributions around 8–12 kb, and barcoded SMRTbell libraries were prepared with the SMRTbell Template Kit. Sequencing was performed on PacBio Sequel (Pacific Biosciences, CA, USA) with 2 h pre-extension and 10 h movie time. Raw sequences were processed in SMRTLink v.8.0 and assembled using HGAP4. Hybrid assembly was performed with Unicycler v.0.4.0 using long- and short-read sequences (15).

Sequencing analysis was done using BacPipe v.1.2.6, a bacterial WGS analysis pipeline (16). The *mgrB* genetic modifications and SNPs were analyzed using CLC Genomics Workbench v.9.5.3 (Qiagen, Hilden, Germany).

Genome annotation was conducted by Prokka (https://github.com/tseemann/prokka) (17), and the annotated fragments were used for insertion sequence detection. Specifically, insertion sequences within genomes were detected by using BLAST-based searching (*E*-value < 10^−5^, identification > 90% match, and coverage > 90%) based on ISfinder database v.2.0 (https://isfinder.biotoul.fr/) (18). Within each genome, IS hits were retrieved. The heatmap was generated with the heatmap package in R. The figure demonstrating the linear comparison between genomes was generated using Easyfig 2.2.3 (19).

The biofilm formation of all strains was determined using an *in vitro* static biofilm assay where optical densities (OD492) were measured and compared with a simultaneously run *Klebsiella quasipneumoniae* reference strain, ATCC700603.

## Data Availability

All sequenced data generated and analyzed in this study were deposited at NCBI under BioProject ID PRJNA948355.

## SUPPLEMENTAL MATERIAL

Supplemental material aac.01574-24-s0001.docx Table S1 to S3.

ASM does not own the copyrights to Supplemental Material that may be linked to, or accessed through, an article. The authors have granted ASM a non-exclusive, world-wide license to publish the Supplemental Material files. Please contact the corresponding author directly for reuse.
