# Supplementary material for: Characterization of genome-wide transpositions induced by colistin exposure in multi-drug-resistant Klebsiella pneumoniae
Source: Antimicrob Agents Chemother. 2025 May 19;69(7):e01574-24. doi: 10.1128/aac.01574-24 (PMC12217459; doi:10.1128/aac.01574-24)
Supplement: Supplemental material — Table S1 to S3. [file aac.01574-24-s0001.docx]

**Supplementary data**

**Supplementary table 1:** Summary of resistance profile of strains used in the study

**Supplementary table 2:** Insertion Sequence (IS) Elements and their copy numbers across strain pairs

IS elements highlighted in red represent additional insertion sequences identified in the colistin-resistant strain that were not present in the colistin-susceptible strain. The IS elements highlighted in bold red represent additional copies of insertion sequences identified in the colistin-resistant strain compared to the colistin-susceptible strain.

**Supplementary table 3:**

Genotypic changes associated with phenotypic emergence of colistin resistance in strain pairs.

No mobile colistin resistance (mcr) gene was identified in any of the strains.
